# Spontaneous Coronary Artery Dissection in Pyridoxine‐Responsive Classical Homocystinuria: Expanding the Arterial Phenotype

**DOI:** 10.1002/jmd2.70145

**Published:** 2026-11-09

**Authors:** Eamon P McCarron

**Affiliations:** ^1^ Adult Inherited Metabolic Disorders Sheffield Teaching Hospitals NHS Foundation Trust Sheffield UK; ^2^ Faculty of Health University of Sheffield Sheffield UK

Dear Editor,

I read with great interest the recent report by Chard et al. [1] describing the first reported case of thoracic aortic dissection in classical cystathionine β‐synthase (CBS) deficiency and proposing that aortic surveillance may be warranted in selected patients. Together with previous reports of coronary and carotid artery dissection [2, 3] their observations add to the growing recognition that the vascular phenotype of CBS deficiency extends beyond its traditional thromboembolic manifestations to include structural arterial disease.

I report a complementary arterial manifestation in a 29‐year‐old man, the first of three children born to non‐consanguineous parents, who presented with a two‐week history of chest pain and left‐sided weakness. The patient had no history of hypertension, dyslipidaemia or other recognised cardiovascular risk factors. Blood pressure, lipid profile, renal function and liver biochemistry were within normal limits, with no known hepatic or renal comorbidities. Electrocardiography demonstrated an anterior ST‐elevation myocardial infarction, and coronary angiography confirmed spontaneous coronary artery dissection (SCAD) of the proximal left anterior descending artery (Figure 1A). Transthoracic echocardiography demonstrated severe left ventricular systolic dysfunction with a large apical thrombus (Figure 1B), and he subsequently underwent thrombectomy for a right middle cerebral artery occlusion. Initial biochemical investigations demonstrated plasma total homocysteine of 311 μmol/L, vitamin B12 of 176 ng/L, folate of 2.8 μg/L and hypermethioninaemia (peak 86 μmol/L), consistent with classical homocystinuria. Molecular analysis identified two pathogenic CBS variants, c.833 T> C (p.Ile278Thr) and c.919G> A (p.Gly307Ser), confirmed to be in trans, establishing compound heterozygous CBS deficiency. Treatment with folic acid, vitamin B12, betaine and subsequently pyridoxine reduced plasma total homocysteine to 16–19 μmol/L and methionine to 22–29 μmol/L, confirming pyridoxine responsiveness. Given the large left ventricular apical thrombus, long‐term anticoagulation with the vitamin K antagonist warfarin was instituted. During 2 years of follow‐up, he experienced no recurrent thromboembolic events, although persistent left ventricular systolic dysfunction required ongoing heart failure management. Cascade family screening subsequently identified previously unrecognised pyridoxine‐responsive CBS deficiency in his sister following an unprovoked pulmonary embolism.

**FIGURE 1:**
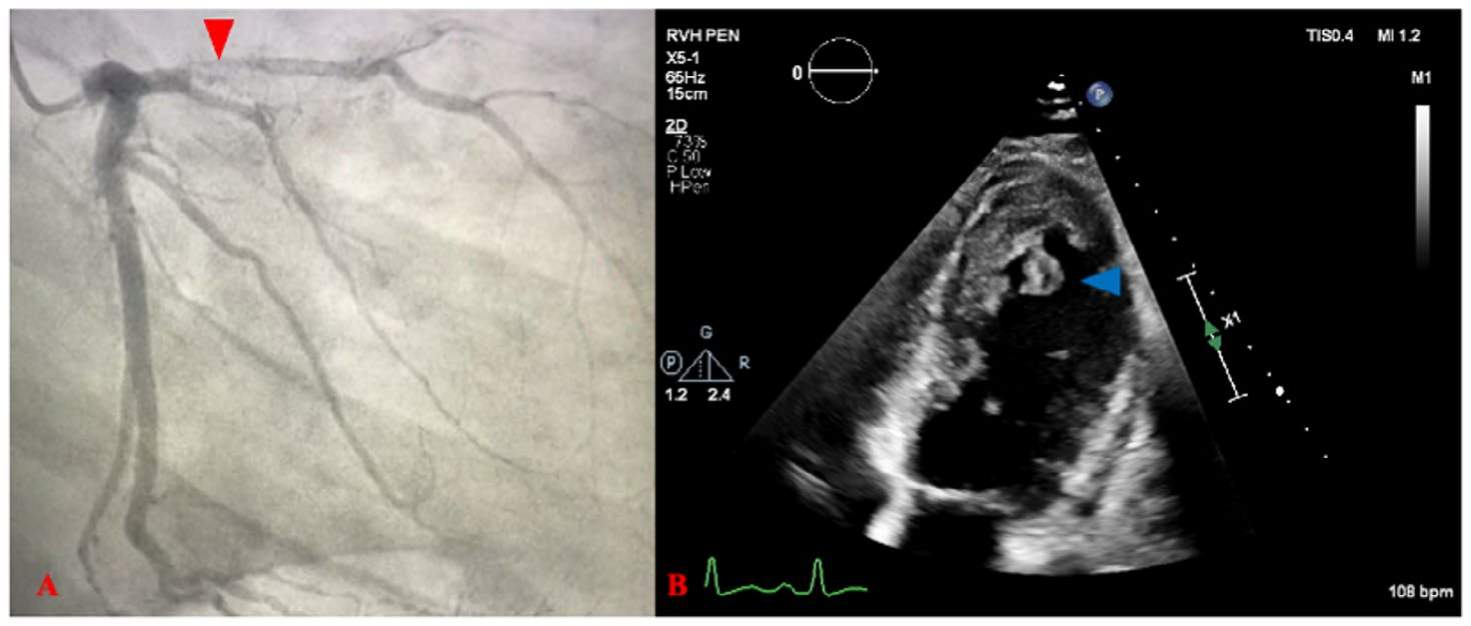
Cardiovascular manifestations at presentation. (A) Coronary angiography demonstrating spontaneous coronary artery dissection (SCAD) of the proximal left anterior descending coronary artery (red arrowhead), presenting as an anterior ST‐elevation myocardial infarction. (B) Transthoracic echocardiography (apical four‐chamber view) demonstrating a large left ventricular apical thrombus (blue arrowhead) associated with severe left ventricular systolic dysfunction.

Although thromboembolism remains the hallmark vascular complication of CBS deficiency, arterial dissections involving the coronary and carotid arteries have previously been reported [2, 3]. Taken together with the report by Chard et al. [1], the present case broadens the recognised arterial phenotype of CBS deficiency and suggests that structural arterial disease has the potential to affect multiple vascular beds. Mechanistic studies demonstrate that chronic hyperhomocysteinaemia impairs endothelial redox homeostasis, promotes oxidative stress and extracellular matrix remodelling, providing biologically plausible mechanisms for arterial wall weakening [4, 5, 6]. In this patient, SCAD occurred at presentation in the setting of profound untreated hyperhomocysteinaemia before excellent biochemical control was achieved with therapy.

Notably, this patient had pyridoxine‐responsive CBS deficiency, a phenotype that is frequently under‐recognised by methionine‐based newborn screening because residual enzyme activity may prevent significant neonatal hypermethioninaemia. Contemporary registry data indicate that approximately 38% of patients exhibit some degree of pyridoxine responsiveness, yet no fully or extremely responsive individuals were identified among patients detected by newborn screening, highlighting the limitations of methionine‐based screening strategies [7, 8].

Although isolated case reports cannot establish causality or justify routine cardiovascular imaging, they broaden the recognised vascular phenotype of CBS deficiency beyond thromboembolism. Prospective multicentre studies incorporating genotype, metabolic control and cardiovascular imaging are needed to determine whether structural arterial disease is more prevalent than currently appreciated. Current international guidelines appropriately emphasise prevention of thromboembolism and optimisation of metabolic control but do not address spontaneous arterial dissection or cardiovascular surveillance, despite recognised connective tissue manifestations of CBS deficiency, including aortic root dilatation [9, 10]. As additional reports of structural arterial disease emerge, future guideline updates may need to consider whether selected patients with CBS deficiency would benefit from targeted cardiovascular assessment and surveillance.

## Funding

The author has nothing to report.

## Consent

Patient consent was obtained.

## Data Availability

Data sharing not applicable to this article as no datasets were generated or analysed during the current study.
